# Applying Brazilian front-of-pack nutrition labeling and PAHO nutritional criteria to digital food advertising: regulatory implications for protecting children and adolescents

**DOI:** 10.1371/journal.pone.0354180

**Published:** 2026-07-17

**Authors:** Juliana de Paula Matos, Breenda Lorranny Santos Gonçalves Araújo, Mariana Ribeiro, Laís Amaral Mais, Paula Martins Horta

**Affiliations:** 1 Department of Nutrition, School of Nursing, Federal University of Minas Gerais, Belo Horizonte, Minas Gerais, Brazil; 2 Department of Nutrition, School of Nursing, Federal University of Minas Gerais, Belo Horizonte, Minas Gerais, Brazil; 3 Center for Epidemiological Research in Nutrition and Health (Nupens), University of São Paulo, São Paulo, São Paulo, Brazil; 4 Brazilian Institute for Consumer Defense, São Paulo, São Paulo, Brazil; 5 Brazilian Institute for Consumer Defense, São Paulo, São Paulo, Brazil; 6 Department of Nutrition, School of Nursing, Federal University of Minas Gerais, Belo Horizonte, Minas Gerais, Brazil; University of Georgia, UNITED STATES OF AMERICA

## Abstract

Front-of-pack nutrition labeling (FoPNL) and restrictions on food advertising are regulatory strategies to support informed dietary choices and reduce exposure to unhealthy food. These measures are often integrated through a policy package, that employs a Nutrient Profile Model (NPM) for the classification of food. In Brazil, however, these policies remain fragmented, and current food advertising regulations do not fully cover all channels, particularly digital media. This study applied the NPM adopted in Brazil’s FoPNL (RDC No. 429/2020 in conjunction with IN No. 75/2020, hereafter RDC 429) and those proposed by the Pan American Health Organization (PAHO) for identifying foods subject to advertising restrictions on social media posts directed at children and adolescents. The sample included posts from ultra-processed food brands/products on Instagram (n = 623), TikTok (n = 257), and YouTube (n = 114). Both NPM were applied to assess non-compliance at the food-item level and at the advertisement level (considering posts containing at least one non-compliant food). At the food-item level, 61.28% of products exceeded at least one item of RDC 429, with the highest prevalence on TikTok (72.63%), while 93.86% were non-compliant according to PAHO, reaching 100% on TikTok and 98.5% on YouTube. At the advertisement level, 49.53% of posts contained at least one non-compliant food under RDC 429, and 74.13% under PAHO, with YouTube showing the greatest potential for restriction (62.16% RDC; 99.10% PAHO). Both models are feasible for regulating digital food advertising: the PAHO provides a robust framework, while RDC 429 offers a strategic starting point given its current implementation in Brazil.

## Introduction

Regulatory measures designed to limit the commercial practices of ultra-processed food corporations are widely recommended to mitigate the health impacts of excessive consumption of these products, particularly the development of noncommunicable diseases (NCD) such as obesity, hypertension, and type 2 diabetes [1,2]. To achieve this objective, such measures should form part of a comprehensive regulatory framework that includes restrictions on advertising directed at children and adolescents, front-of-pack nutrition labeling (FoPNL), taxation of unhealthy products, and controls on the availability of unhealthy foods in institutional settings. Collectively, these actions reshape the food environment in which people purchase and consume food, simultaneously influencing millions of individuals and contributing to the prevention of NCD [3].

Among these regulatory approaches, restrictions on food advertising and the implementation of a FoPNL stand out due to their potential both to reduce population exposure to the marketing of unhealthy foods and to increase awareness of the risks associated with their excessive consumption. Advertising restrictions have been recommended by the World Health Organization (WHO) since 2010, with updated guidance issued in 2023 urging governments to address both the extent of exposure to such marketing and its persuasive power [4]. With respect to FoPNL, WHO recommends government-led regulation as a means of fostering more informed dietary choices by highlighting excessive levels of critical nutrients through warning systems [5].

Although distinct in their mechanisms, these measures are frequently integrated through the adoption of a common Nutrient Profile Model (NPM), which serves as the criterion to determine which foods should be subject to regulation. By definition, NPM are science-based tools for classifying or ranking foods according to their nutritional composition, grounded in health promotion and disease prevention. Their application enables the standardized identification of foods with excessive levels of nutrients of concern such as sugars, sodium, and fats, thereby ensuring greater coherence and effectiveness across regulatory measures [6]. In the Region of the Americas, the Pan American Health Organization (PAHO) has developed a NPM to guide governments in identifying unhealthy foods products and implementing public policies to discourage their consumption. This model defines foods as unhealthy when they contain excessive amounts of free sugars, total fats, saturated fats, trans fats, and sodium, as determined by its NPM [7].

Latin American countries provide relevant examples of how such regulatory packages can be implemented, particularly regarding the simultaneous and coordinated application of advertising restrictions and FoPNL. In Argentina, for instance, Law No. 27,642 of 2021 prohibits the advertising of foods bearing FoPNL for high contents of sugars, sodium, total fats, and saturated fats across multiple media outlets and in school settings [8]. In Chile, Law No. 20,606 of 2012 imposes restrictions on advertisements for foods high in sugars, sodium, and fats, identified through NPM, during peak child-audience hours, in digital media, and in child-focused spaces such as schools [9].

In contrast, Brazil regulates advertising restrictions and FoPNL separately and independently. With respect to advertising, existing legislation prohibits misleading advertising (capable of deceiving consumers) and abusive advertising (particularly when directed at children), as established in the Consumer Defense Code and reinforced by Resolution No. 163 of the National Council for the Rights of Children and Adolescents (CONANDA) [10,11]. Although applicable to food, these provisions are not specifically designed for food advertising. Monitoring studies in Brazil reveal high levels of non-compliance by the food industry, which continues to promote unhealthy products, particularly through child-targeted marketing in both traditional [12] and digital media [13].

Brazilian FoPNL is regulated by Collegiate Board Resolution (RDC) No. 429/2020 [14] and Normative Instruction (IN) No. 75/2020, both issued by the National Health Surveillance Agency (ANVISA) on October 8, 2020 [15]. These regulations mandate the use of a magnifying-glass symbol to identify high levels of nutrients of concern (added sugars, sodium, and saturated fats) in processed and ultraprocess foods (Brasil, 2020a). However, the NPM adopted are not aligned with internationally recommended NPM, establish thresholds regarded as insufficiently strict, and were not validated prior to implementation, thereby limiting their effectiveness and underscoring the need for refinement [16].

In light of this fragmented regulatory framework and the absence of specific legislation on food advertising, integrating FoPNL, already implemented in Brazil, with restrictions on the marketing of unhealthy foods emerges as a strategic approach to strengthening national efforts to combat NCD and to improve the food environment. In this regard, the nutritional parameters applied in labeling could also serve as a basis for coordinating advertising restrictions across different communication channels and media platforms.

Progress in this direction is particularly important given the evolving nature of digital advertising strategies. In digital environments, both exposure to and the persuasive power of advertising are intensified, with direct targeting of specific audiences, including children and adolescents, and increased frequency and intensity of exposure, which render these practices more difficult to detect and regulate compared with traditional media [4]. Social media platforms such as TikTok, Instagram, and YouTube are especially influential, given their broad reach and widespread use among children and adolescents. These platforms employ algorithms that personalize content and extend screen time, thereby increasing the likelihood of frequent exposure to ultra-processed food advertising [17].

The objective of this study is to apply the NPM criteria adopted in Brazilian FoPNL and those proposed by PAHO to identify foods subject to advertising restrictions in Brazil, drawing on content disseminated by the ultra-processed food industry targeting children and adolescents on Brazilian social media. It is important to note that this study does not aim to evaluate the effectiveness of FoPNL or advertising restrictions. Rather, it provides a structured descriptive analysis of how different regulatory nutrient profile models may be operationalized to assess and potentially coordinate restrictions on digital food advertising targeting children and adolescents.

## Methods

### Sample selection and characterization

The identification of brands/products, social media platforms and advertisements eligible for the study was guided by the WHO/Europe CLICK framework, which provides structured guidance for monitoring food marketing across multiple domains [18]. The process was conducted in two stages: (i) defining the set of products/brands and (ii) selecting social media platforms and the corresponding advertisements.

In the first stage, a database compiled from five of the largest retail food chains in São Paulo, covering approximately 70% of branded products available in the city, was consulted [19]. Food labels from this database were systematically screened to identify communication elements targeting children and adolescents, based on the criteria described by Borges et al. (2022) [19]. These elements included promotional characters, mascots, references to health or energy, sports-related themes, prizes, and imagery featuring children or fruits and vegetables. This screening yielded 1,054 products displaying at least one child-directed marketing strategy. From this total, only products classified as ultra-processed according to the NOVA classification system [20] were retained, resulting in a subsample of 724 products/brands.

YouTube, Instagram, and TikTok were selected as the social media platforms for this study, based on the 2024 TIC Kids Online Brazil survey, a nationally representative study supported by UNESCO, UNICEF, and the Economic Commission for Latin America and the Caribbean (CEPAL). This survey identified these platforms as the most frequently used among Brazilian children and adolescents aged 9–17 years [21].

Subsequently, the official social media accounts of the 724 products/brands were identified for the three selected platforms. Among these, 604 had active accounts on at least one of the platforms. The content of these pages was analyzed using the same child-targeted criteria applied to food packaging. After excluding supermarket chains, companies with broad product portfolios not specifically aimed at children, and duplicate product lines, 33 unique brand pages remained. From each page, 20 posts published in 2023 were randomly sampled, in accordance with CLICK framework recommendations. The final sample comprised 994 posts: 623 from Instagram, 257 from TikTok, and 114 from YouTube.

### Food identification and classification

All industrialized foods displayed in the selected advertisements were identified, and their nutritional information was collected from food labels, brand websites, or retailer websites between August and October 2024. Foods for which nutritional information was unavailable at the time of data collection were excluded (n = 56). In advertisements featuring multiple foods, only items representative of the advertised brand were considered. For example, in an advertisement for a juice brand that also displayed cakes or fruit bars, the juice was classified as eligible. Conversely, when all foods depicted were representative of the brand, for instance, a chocolate brand advertisement featuring various types of chocolates – all items were included in the analysis. The final analytical sample comprised of 1,498 foods.

Subsequently, foods were categorized according to the CLICK framework recommendations: (a) candies and chewing gum (29.64%); (b) breads, cakes, and cookies (23.36%); (c) snacks (15.49%); (d) dairy and chocolate beverages (11.48%); (e) sweets and chocolates (7.34%); (f) juices and soft drinks (6.07%); (g) breakfast cereals (4.14%); (h) ultra-processed cheeses (2.40%); and (i) ready-to-eat meals (0.07%) (Tatlow-Golden et al. 2021).

### Nutritional criteria applied to promoted foods

To determine which foods advertised in the selected posts would be subject to restrictions, two NPM were applied: the ANVISA Resolution RDC No. 429/2020 in conjunction with IN No. 75/2020 (hereafter jointly referred to as RDC 429), and the PAHO NPM (hereafter referred to as PAHO). A comparative table outlining the specific features of each model is presented below (Table 1). It should be emphasized that, in the application of the PAHO, only added sugars were considered, as Brazilian nutrition labeling does not provide information on free sugars, which is required by the model.

**Table 1:** Comparison of RDC 429 and PAHO Nutrient Profile Model criteria for foods.

Nutrient	RDC 429**	PAHO
Added sugars*	≥ 15 g of added sugars per 100 g of food or ≥ 7.5 g per 100 mL of food	≥ 10% of total energy from free sugars
Other sweeteners	NA	Any amount of other sweeteners
Total fats	NA	≥ 30% of total energy from total fats
Saturated fats	High in saturated fats when ≥ 6 g per 100 g of food or ≥ 3 g per 100 mL of food	≥ 10% of total energy from saturated fats
Trans fats	NA	≥ 1% of total energy from trans fats
Sodium	High in sodium when ≥ 600 mg per 100 g of food or ≥ 300 mg per 100 mL of food	≥ 1 mg of sodium per 1 kcal

Note: RDC 429 – RDC No. 429/2020 and Normative Instruction No. 75/2020; PAHO – Pan American Health Organization.*In all analyses, only added sugars were considered, although PAHO applies to free sugars content. **For products requiring reconstitution, the nutritional information was based on the product as prepared for consumption.

### Data analysis

Absolute and relative frequencies were used to describe the adequacy of foods according to the two NPM. Classification was performed for each individual criterion of the models and globally, considering a food item as non-compliant if it exceeded at least one criterion. This analysis was conducted at the food-item level for the entire set of advertised foods and stratified by food categories.

To assess adequacy at the advertisement level, advertisements featuring more than one food were classified as non-compliant if at least one of the foods exceeded any of the nutritional criteria defined by the models. Permitted advertisements included both those that did not feature foods violating the NPM criteria and brand-only advertisements, i.e., advertisements without any foods present (n = 216 advertisements).

All analyses were conducted using a 95% confidence interval, with differences considered significant when confidence intervals did not overlap. Analyses were performed for the overall sample and stratified by social media platform. Statistical analyses were carried out using Stata version 14.0.

The dataset was constructed from content that was publicly available across the selected platforms during the study period. Data were collected through a systematic manual procedure based on predefined inclusion criteria. Only institutional or brand-related content was included, and no personal or private user information was accessed. All data collection and analytical procedures were conducted in accordance with the terms of service and conditions of use of the respective platforms.

## Results

At the food-item level, the application of the RDC 429 indicated that 61.28% of foods advertised in social media posts exceeded at least one of the model’s parameters. Among the platforms analyzed, the highest prevalence of non-compliance was observed on TikTok (72.63%), followed by Instagram (59.88%) and YouTube (49.06%). When applying the PAHO, 93.86% of the foods exceeded at least one critical nutrient threshold, with the highest proportions on TikTok (100%) and YouTube (98.50%) (Table 2).

**Table 2:** Application of the RDC 429/2020 and PAHO NPMs to foods advertised on Brazilian social media.

Nutrient criteria	General				Instagram				TikTok				YouTube			
	RDC 429		PAHO		RDC 429		PAHO		RDC 429		PAHO		RDC 429		PAHO	
	%	95% CI	%	95% CI	%	95% CI	%	95% CI	%	95% CI	%	95% CI	%	95% CI	%	95% CI
Added sugars^*^	45.99	43.48-48.53	62.62	60.13-65.05	44.64	41.30-48.03	60.00	56.64-63.27	56.01	51.04-60.86	71.87	67.19-76.11	35.58	30.05-41.52	57.30	51.28-63.12
Total fats	NA	NA	39.52	37.07-42.02	NA	NA	37.74	34.51-41.07	NA	NA	42.71	37.88-47.68	NA	NA	40.45	34.71-46.46
Saturated fats	23.77	21.67-25.99	35.78	33.39-38.25	20.95	18.33-23.84	30.36	27.34-33.56	28.64	24.37-33.34	42.71	37.88-47.68	25.47	20.59-31.05	42.70	36.88-48.72
Trans fats	NA	NA	3,60	2.77-4.68	NA	NA	2.38	1.54-3.66	NA	NA	5.63	3.73-8.40	NA	NA	4.49	2.56-7.76
Sodium	9.21	7.85-10.79	21.43	19.42-23.58	10.71	8.79-13.00	23.45	20.70-26.44	6.91	4.77-9.89	19.18	15.57-23.40	7.87	5.18-11.77	18.35	14.14-23.47
Sweeteners	NA	NA	14,82	13,11-16,71	NA	NA	11.07	9.12-13.38	NA	NA	16.11	12.78-20.10	NA	NA	24.72	19.90-30.26
**Adequate**	**38**.**72**	**36**.**28-41**.**21**	**6**.**14**	**5**.**03-7**.**48**	**40**.**12**	**36**.**85-43**.**48**	**10**.**48**	**8**.**58-12**.**74**	**27**.**37**	**23**.**17-32**.**01**	**0**.**00**	**–**	**50**.**94**	**44**.**94-56**.**91**	**1**.**50**	**0**.**56-3**.**93**
**Non-compliance with at least one criterion**	**61**.**28**	**58**.**79-63**.**72**	**93**.**86**	**92**.**52-94**.**97**	**59**.**88**	**56**.**52-63**.**15**	**89**.**52**	**87**.**26-91**.**42**	**72**.**63**	**68**.**00-76**.**83**	**100**.**0**	**–**	**49**.**06**	**43**.**09-55**.**06**	**98**.**50**	**96**.**07-99**.**44**

Note: Missing data (a: n = 87). *In all analyses, only added sugars were considered, although PAHO applies to free sugars content.

Added sugars were the nutrient with the highest prevalence of non-compliance across both NPM, regardless of the platform. Using the RDC 429, 45.99% of foods exceeded the cut-off for added sugars, with the highest proportion on TikTok (56.01%) and similar values on Instagram (44.64%) and YouTube (35.58%). According to the PAHO, non-compliance for added sugars was even more pronounced, affecting 62.62% of foods analyzed, with minor differences across platforms (Instagram: 60.0%; TikTok: 71.87%; YouTube: 57.30%) (Table 2).

At the food-item level and stratified by food category, RDC 429 indicated that 100% of ultra-processed cheeses promoted on posts exceeded at least one nutrient criterion, followed by breakfast cereals (90.32%), breads, cakes, and cookies (88.29%), sweets and chocolates (77.27%), and candies and chewing gum (67.57%). When stratified by social media, Instagram showed a similar pattern, with the same five categories exceeding approximately 65% non-compliance. On TikTok, 100% of ultra-processed cheeses, breakfast cereals, sweets and chocolates, and snacks exceeded at least one RDC 429, while breads, cakes, cookies, and snacks reached 97.73% non-compliance. On YouTube, the highest non-compliance rates were observed in breakfast cereals (100%), sweets and chocolates (100%), breads, cakes, and cookies (83.67%), and ultra-processed cheeses (100%) (Table 3).

**Table 3:** Food categories advertised on Brazilian social media according to non-compliance with at least one nutrient criterion under the RDC 429/2020 and PAHO NPM.

Food categories	Non-compliance with at least one criterion															
	General				Instagram				TikTok				YouTube			
	RDC 429		PAHO		RDC 429		PAHO		RDC 429		PAHO		RDC 429		PAHO	
	%	95% CI	%	95% CI	%	95% CI	%	95% CI	%	95% CI	%	95% CI	%	95% CI	%	95% CI
Candies and chewing gum	67.57	63.06-71.77	99.32	97.92-99.78	78.57	72.48-83.62	98.57	95.64-99.54	69.62	61.97-76.32	100	–	32.89	23.22-44.27	100	–
Dairy and chocolate beverages	6.40	3.57-11.2	100	–	7.14	2.97-16.22	100	–	5.88	1.87-16.99	100	–	5.88	1.87-16.99	100	–
Breakfast cereals	90.32	79.96-95.62	85.48	74.27-92.31	79.31	60.13-90.69	68.97	49.54-83.41	100	–	100	–	100	–	100	–
Sweets and chocolates	77.27	68.47-84.18	98.18	92.97-99.55	64.79	52.84-75.13	97.18	89.18-99.31	100	–	100	–	100	–	100	–
Breads, cakes, and cookies	88.29	84.47-91.26	96.29	93.70-97.83	85.45	80.01-89.59	95.77	92.05-97.79	97.73	91.26-99.44	100	–	83.67	70.39-91.7	91.84	79.96-96.94
Ultra-processed cheeses	100	–	100	–	100	–	100	–	100	–	100	–	100	–	100	–
Snacks	40.52	34.37-46.98	86.64	81.61-90.45	26.14	19.75-33.74	79.74	72.56-85.42	100	–	100	–	32.43	19.22-49.19	100	–
Juices and soft drinks	29.67	21.15-39.88	62.64	52.23-71.99	32.89	23.13-44.41	55.26	43.78-66.21	10.00	1.21-50.25	100	–	20.00	2.04-74.97	100	–
Ready-to-eat meals	0	–	100	–	0	–	100	–	0	–	0	–	0	–	0	–

Under the PAHO, non-compliance rates were higher across categories. Except for juices and soft drinks (62.64%), all food categories exceeded at least one nutrient threshold in over 85% of products: pre-prepared meals (100%), ultra-processed cheeses (100%), dairy and chocolate beverages (100%), candies and chewing gum (99.32%), sweets and chocolates (98.18%), breads, cakes, and cookies (96.29%), snacks (86.64%), and breakfast cereals (85.48%). On Instagram, six of these eight categories had non-compliance ≥95%. On TikTok, all analyzed categories reached 100% non-compliance, while on YouTube, all categories except pre-prepared meals (91.84%) reached 100% non-compliance (Table 3).

At the advertisement level, 49.53% of posts promoted at least one food exceeding a critical nutrient threshold and would therefore be eligible for restriction under RDC 429, while 74.13% would be restricted under PAHO. Among social media platforms, applying RDC 429, YouTube showed the highest proportion of restricted advertisements (62.16%), followed by Instagram (45.39%). Under PAHO, nearly all YouTube advertisements were restricted (99.10%), exceeding restriction levels observed on TikTok and Instagram (Table 4).

**Table 4:** Restriction of advertisements on Brazilian social media according to non-compliance with at least one nutrient criterion under RDC 429/2020 and PAHO NPM.

Posts	RDC 429		PAHO	
	%	CI 95%	%	CI 95%
General	49.53	46.35-52.71	74.13	71.25-76.82
Instagram	45.39	41.39-49.45	68.09	64.19-71.75
TikTok	53.54	47.37-59.61	77.17	71.58-81.93
YouTube	62.16	52.76-70.73	99.10	93.82-99.87

## Discussion

This study enabled the analytical operationalization of the potential integration between regulatory measures, specifically FoPNL criteria and restrictions on food advertising, within the still underexplored context of digital food advertising environments. The comparison of results obtained using the RDC 429, applied in Brazil’s FoPNL, with those from the PAHO in the context of food advertising on Brazilian social media revealed substantial differences in the potential scope of restrictions. At the food-item level, 61.28% of foods were classified as exceeding at least one RDC 429, whereas 93.86% were considered excessive under the PAHO. At the post level, 49.53% of advertisements would be restricted according to RDC 429, compared to 74.13% under the PAHO. In both models, added sugar content was the most common criterion for non-compliance. Furthermore, stratified analysis by social media platform indicated that, at the food-item level, TikTok showed the highest frequency of products subject to restriction, whereas at the advertisement level, YouTube ads were the most likely to be regulated.

The greater permissiveness observed in RDC 429 arises from structural characteristics of its NPM, particularly when compared with those of the PAHO. Key limitations of RDC 429 include the omission of non-sugar sweeteners as a restriction criterion, which may incentivize product reformulation through partial or total substitution of added sugars with non-sugar sweeteners to maintain sweetness. However, this is undesirable, as evidence from prospective cohort studies, summarized by the WHO, indicates that the consumption of such additives may be associated with potential long-term harm, including an increased risk of obesity, type 2 diabetes, cardiovascular diseases, and mortality [22]. Another important limitation of RDC 429 is the use of lenient thresholds for regulated nutrients. The limits established for sodium, added sugars, and saturated fats in Brazil’s FoPNL are more permissive than those of other international models, such as the PAHO NPM. These less stringent cutoffs reduce the model’s ability to identify foods with high concentrations of critical nutrients, potentially underestimating the proportion of products that should be subject to regulation.

Compared to other countries in the Region of the Americas, there is considerable diversity in the nutrients regulated by FoPNL. Argentina, Mexico and Chile include sugars, saturated fats, sodium, and calories. Among these countries, Argentina and Chile prohibit advertising of products that carry at least one FoPNL warning label, while Mexico restricts such advertising specifically when directed at children [23]. Overall, the results highlight the diversity of approaches adopted in the Region of the Americas, emphasizing differences in nutrient selection and thresholds, as well as the significant progress achieved in countries such as Argentina, Mexico, and Chile, which combine FoPNL with restrictions on advertising directed at children [23].

Compared to these countries, Brazil’s FoPNL regulation covers a smaller number of nutrients and adopts relatively more lenient thresholds. During the regulatory process, scientific guides and evidence were presented indicating the possibility of adopting more robust cut-offs for critical nutrients, as well as additional regulatory strategies. Although these submissions included many other nutrients and measures that could have been regulated, the final criteria reflected significant influence from the regulated sector, which exerted pressure on the regulatory agency [16]. Despite these limitations, the nutrients currently regulated remain strategically important. First, they are concentrated in ultra-processed foods, aligning with the Brazilian Dietary Guidelines, which recommend limiting consumption of such products, even though many were excluded due to the thresholds adopted [1]. Second, they represent a key strategy to address the burden of chronic diseases in Brazil, with prevalence rates of arterial hypertension at 29.7%, diabetes at 12.9%, and obesity at 25.7% [24].

In both models, RDC 429 and PAHO, added sugar content was the leading criterion for identifying foods with an inadequate nutrient composition. Added sugars refer to sugars and syrups incorporated into foods and beverages during processing, preparation, or meals, including compounds such as white sugar, high-fructose corn syrup, honey, fruit juice, and fruit juice concentrates, while excluding naturally occurring sugars such as lactose in milk and sucrose or fructose in fruits [25]. Excessive consumption of added sugars is strongly associated with increased risk of weight gain, obesity, type 2 diabetes, cardiovascular diseases, and dental caries. Moreover, added sugars contribute to poor-quality diets, often linked to ultra-processed food consumption [25]. Although both models were highly sensitive to this criterion, their application differs. RDC 429 establishes separate thresholds for added sugars in liquid foods (≥7.5 g/100 mL) and in solid or semi-solid foods (≥15 g/100 g), resulting in liquid product categories being evaluated under more permissive criteria. In contrast, the PAHO recommends the application of a single threshold for all foods, regardless of consistency, providing a more consistent and potentially more protective assessment. Furthermore, it should be emphasized that the nutrient regulated by the PAHO is free sugars; however, in the present analysis, these were not considered due to the unavailability of this information in Brazilian nutrition labeling, and only added sugars were used. Since free sugars comprise a broader category than added sugars, this approach likely underestimated the proportion of products that would exceed the PAHO threshold. Therefore, the difference observed between the PAHO criteria and RDC 429 may be even greater than that identified in the present study, reinforcing the greater stringency and sensitivity of the PAHO model in identifying products with excessive levels of critical nutrients.

Despite discrepancies between models, both identified ultra-processed cheeses, breakfast cereals, sweets and chocolates, breads, cakes and cookies, and candies and chewing gum as foods with nutritionally risky profiles. Although, not all models flagged the same nutrients as exceeding the established thresholds. According to the 2017–2018 Household Budget Survey, these ultra-processed foods represent 26.7% of total caloric intake among adolescents aged 10–18 years in Brazil [26].

When analyzing results by social media platform, methodological particularities became evident. At the food-item level, TikTok exhibited the highest frequency of products exceeding nutrient thresholds, whereas at the advertisement level, YouTube advertising were the most likely to be subject to regulation. This discrepancy can be explained by the distribution of products within advertising content: in our sample, YouTube posts contained, on average, more food items per post compared with TikTok (3.65 vs. 3.08, respectively). Because an advertisement is classified as non-compliant if at least one of its products exceeds the nutrient criteria, a higher number of products per post increases the probability that a given advertisement will be restricted. This explains why the proportion of restricted advertisements was higher on YouTube, even though the proportion of non-compliant products was higher on TikTok. These findings highlight that both YouTube and TikTok are particularly sensitive to regulatory parameters, especially given the profile of foods promoted on these platforms.

In Brazil, ultra-processed food advertising on YouTube is substantial, representing 93.8% of food advertisements in popular videos across the 25 most-watched children’s channels. Additionally, videos containing food advertisements tend to receive more “likes” than those without such content [13]. YouTube’s greater potential for restriction is attributable to platform-specific characteristics, including longer video formats and the use of persuasive strategies such as unboxings and product reviews, primarily featuring ultra-processed foods. These strategies increase exposure and product appeal, enhancing the likelihood that advertised foods will be classified as restricted under the NPM.

Regarding TikTok, although evidence on unhealthy food advertising in Brazil is still limited, a study of popular influencer profiles in the United States analyzed 1,360 videos featuring at least one food product, which collectively amassed over 9 million views and more than 1 million likes each. Moreover, when branded products were presented, most posts did not include any disclosure of brand partnerships [27]. These findings suggest that TikTok, similarly to YouTube, has considerable potential for promoting ultra-processed foods, underscoring the need for monitoring and regulation of food advertising across emerging social media platforms.

The evidence from this study indicates that it is feasible to align restrictions on food advertising targeting children and adolescents with the nutritional criteria established in Brazil’s FoPNL, using Brazilian social media as the analytical context. While results demonstrate the technical superiority of the PAHO it is important to recognize the strategic and normative role of RDC 429 in the local context. As an already implemented regulation, RDC 429 provides a relevant starting point and a potential facilitator for future adaptations and improvements, including alignment with the more restrictive and protective criteria recommended by international organizations such as PAHO.

In practical terms, advertising restrictions based on the RDC 429 could include: (i) limiting the use of strategies that directly target children and adolescents, such as child characters, celebrities, child-oriented language, animations, and promotional games; and (ii) restricting exposure to advertisements not explicitly aimed at this audience but which may appear in spaces where children and adolescents are potentially present; (iii) exaggerated sensory appeals, such as images emphasizing texture, flavor, or extreme pleasure, which may reinforce impulsive and non-conscious consumption of foods with inadequate nutritional composition; (iv) sponsorship of events, programs, or cultural, sports, or educational content by brands or companies whose products exceed established nutrient limits; (v) the use of gifts, contests, or promotions linked to the consumption of inadequate foods; and (vi) restrictions on associating nutritionally inadequate products with social or environmental causes, thereby promoting a socially responsible or environmentally friendly brand image.

Although the present study focused on digital food advertising, the discussions regarding advertising restrictions raised herein may also be applicable to traditional media, particularly television. In Brazil, a study conducted on the three most popular free-to-air television channels found that 91% of food advertisements featured ultra-processed foods, with soft drinks, alcoholic beverages, and fast-food meals being the most frequently promoted products [12]. Furthermore, these products were advertised using a variety of marketing strategies, including persuasive techniques specifically directed at children and adolescents [28]. These findings suggest that the promotion of foods with unhealthy nutritional profiles is not restricted to digital environments, but rather reflects a broader systemic issue across different media channels. Nevertheless, digital platforms introduce additional challenges related to content personalization, algorithmic targeting, and the integration of advertising with organic content, which may further increase exposure and complicate the monitoring and protection of children and adolescents.

In this context, digital environments may require additional regulatory approaches tailored to their specific advertising dynamics. Specifically in the digital context, these limitations could further include, following WHO guidance [17]: (i) prohibition of algorithmic targeting promoting such products to vulnerable audiences, especially children and adolescents; (ii) limitation on the use of digital influencers or celebrities with significant child or adolescent followings; (iii) prohibition of user-generated content sponsored or incentivized by the industry, such as videos, posts, or viral challenges paid for or encouraged by brands, which promote nutritionally inadequate foods under the guise of organic content, making it difficult to identify as advertising; and (iv) restriction of gamification techniques, including games, challenges, or playful interactions that promote or encourage consumption of nutritionally inadequate foods.

Beyond these recommendations based on nutritional criteria, brand advertising itself should also be considered, as certain ultra-processed products may not meet the nutrients for restriction, but still employ advertising techniques and can still warrant regulatory restrictions. To address this limitation, guidance has been proposed to classify brands based on overall healthiness, not solely on individual products [29]. A recent systematic review reported that brand advertising of foods, beverages, and alcohol may influence consumer preferences, choices, and purchase intentions, although the meta-analysis did not identify a significant effect on actual consumption [30].

While the present study contributes to advancing the regulatory agenda on food advertising, certain limitations must be noted: the nutritional information for identified foods was collected in 2024, so reformulations may have occurred after full implementation of RDC 429. Additionally, only advertisements from official corporate profiles were evaluated, excluding user-generated content. Another limitation is that, due to the lack of information on free sugars in Brazilian nutrition labeling, only added sugars were considered in the application of the PAHO NPM. Consequently, the results may underestimate the number of products exceeding the free sugar threshold, and the actual proportion of products subject to restriction would likely be higher if total free sugars could be assessed. Future research could build on this work by expanding the analysis to other digital environments and by conducting simulations to evaluate the potential impact of coordinated FoPNL and advertising restrictions on individuals of different age groups across various scenarios.

Nonetheless, these limitations do not preclude the conclusion that integrating FoPNL and food advertising within a single regulatory measure represents a feasible and promising strategy to enhance protection, particularly for children and adolescents in digital environments, requiring, however, continued regulatory improvements. It is important to note that digital food advertising differs substantially from traditional media, being highly personalized and targeted. These characteristics pose unique enforcement challenges and highlight the need for adaptive regulatory strategies to ensure the effective protection of children and adolescents. Effective implementation must also consider potential opposition strategies from the industry, which frequently seeks to delay regulatory proposals, generate controversy, discredit proposed actions, and question their effectiveness [31].
